# Efficacy of obstetrics forceps and vacuum extractor to assist during vaginal delivery: systematic review and meta-analysis

**DOI:** 10.61622/rbgo/2025rbgo67

**Published:** 2025-10-21

**Authors:** Rakhi Gaur, Kalpana Thakur, Navjot Kaur, Rajan Kumar, Vipin Patidar, Priyanka Rai

**Affiliations:** 1 All India Institute of Medical Sciences College of Nursing Deoghar Jharkhand India College of Nursing, All India Institute of Medical Sciences, Deoghar, Jharkhand, India.; 2 All India Institute of Medical Sciences College of Nursing Guwahati Assam India College of Nursing, All India Institute of Medical Sciences, Guwahati, Assam, India.; 3 All India Institute of Medical Sciences College of Nursing Bhatinda Punjab India College of Nursing, All India Institute of Medical Sciences, Bhatinda, Punjab, India.; 4 All India Institute of Medical Sciences Department of Paediatrics Deoghar Jharkhand India Department of Paediatrics, All India Institute of Medical Sciences, Deoghar, Jharkhand, India.; 5 All India Institute of Medical Sciences Department of obstetrics and gynaecology Deoghar Jharkhand India Department of obstetrics and gynaecology, All India Institute of Medical Sciences, Deoghar, Jharkhand, India.

**Keywords:** Delivery obstetric, Obstetrical forceps, Caesarean section, Vacuum extraction obstetrical

## Abstract

**Objective:**

When spontaneous vaginal delivery is not feasible, Instrument-assisted vaginal delivery (IVD) is utilized, which often involves the use of vacuum extractors (VE) and obstetric forceps. This study intent to evaluate the risks and benefits associated with utilizing obstetric forceps vs vacuum extractors for IVD, focusing on maternal and neonatal outcomes.

**Methods:**

Following PRISMA guidelines, a full literature search was done in PubMed, Embase, and Google Scholar until April 2024. The review examined randomized controlled trials (RCTs) that compared forceps and vacuum extractors. Two writers extracted the data and assessed its quality separately, with a third resolving any inconsistencies. The statistical study was conducted using R software, with a random-effects model used to quantify risk differences and evaluate heterogeneity.

**Results:**

Seven RCTs, totalling 2,299 individuals, were included. A meta-analysis revealed that forceps significantly increased the incidence of perineal tears (Risk difference = 0.08, 95% CI 0.02-0.13) and vaginal injuries (Risk Difference = 0.12, 95% CI 0.05-0.19). Vacuum extractors were associated with an increased risk of infant cephalohematoma (Risk Difference = −0.06, 95% CI −0.08, −0.04). There was no significant difference in maternal anaesthesia required or failure to accomplish vaginal delivery with the intended instrument.

**Conclusion:**

Obstetric forceps are more likely to cause maternal perineal tears and vaginal injuries, whereas vacuum extractors increase the likelihood of neonatal cephalohematoma. Both methods have comparable anaesthetic needs and success rates for vaginal birth. The clinical scenario ought to guide the choice of instruments, with an emphasis on risk minimization through proper training.

PROSPERO: CRD42024577839.

## Introduction

In specific situations, a normal vaginal delivery may not be feasible or permitted due to various reasons. In such cases, assistance through instruments becomes necessary for childbirth. Two commonly employed methods of instrumental delivery in contemporary obstetric practice include the vacuum extractor (VE) and obstetric forceps.^(1)^ Instrumental vaginal delivery (IVD) plays an important role in expediting or facilitating vaginal delivery. This approach involves utilizing vacuum-assisted devices and/or forceps to aid in the delivery of a fetus. It provides an alternative means to achieve vaginal delivery in appropriately selected cases, thereby decreasing maternal morbidity associated with factors such as blood loss and prolonged hospital stays, which are often consequences of caesarean sections.^(2)^

The practice of instrumental delivery, considered an art, is diminishing and faces the risk of extinction in the foreseeable future, as an increasing number of obstetricians opt for caesarean sections.^(3)^ The prevalence of caesarean sections in India has risen from 17.2% in 2016 to 21.5% in 2021.^(4)^ Notably, instrumental vaginal delivery (IVD) can serve as an alternative to caesarean delivery, helping to avoid associated morbidities like haemorrhage, infection, and complications in subsequent pregnancies. The primary objective of antenatal care is to ensure the optimal health of both the mother and the neonate, underscoring the importance of emphasizing the significance of instrumental vaginal deliveries. While IVD offers benefits, it is essential to acknowledge potential maternal complications such as third- and fourth-degree perineal lacerations, increased blood loss, and vaginal injuries. Neonatal risks associated with IVD include cephalhematoma, haemorrhage, nerve injury, skull fracture, and scalp injury.^(3,5)^

Forceps are a frequently employed tool to assist in vaginal childbirth. Compared to vacuum-assisted delivery, forceps-assisted delivery exhibits a lower failure rate but is linked to a higher occurrence of maternal pelvic floor trauma and a notable decrease in pelvic floor muscle strength.^(6)^ On the contrary, vacuum-assisted vaginal delivery does not indicate links with prolapse or diminished pelvic floor muscle strength but does present an increased incidence of cephalhematoma.^(4,7)^ Forceps involve applying pulling force at the base of the skull, whereas ventouse achieves head extraction through scalp traction via suction. Traditionally, the selection between these two possibilities is often influenced by established practices and training.^(8)^

The reintroduction of this technique holds promise in emergency obstetric care. To uphold instrumental vaginal delivery as a secure alternative to caesarean delivery, it is crucial to provide trainees with education and experience to attain proficiency, while healthcare providers must maintain ongoing experience to sustain their skills. While observational studies extensively detail maternal and offspring outcomes in forceps-assisted births and vacuum assisted births, there is limited evidence from randomized trials. The available evidence is insufficient to favor forceps-assisted interventions over vacuum-assisted births. With a well-defined research question in focus, this systematic review was crafted to scrutinize all randomized clinical trials that compare the risks and benefits of using obstetric forceps against vacuum extractor during childbirth in terms of maternal and foetal outcomes.

## Methods

What are the risks and benefits of using obstetric forceps compared to a vacuum extractor during instrument-assisted vaginal delivery? This question aims to highlight the effectiveness of these tools in assisted vaginal births.

Researchers conducted a comprehensive review of the available literature on the relevant issue. The review was done systematically in accordance with the Preferred Reporting Items for Systematic Reviews and Meta-Analyses (PRISMA) criteria (see supplementary file S1).^(9)^ The study protocol has been registered with PROSPERO under the number CRD42024577839.

Literature review was performed using optimal search terms on PubMed, Embase, and Google Scholar. The first search was conducted until February 2024, with a subsequent revision in April 2024. Each database was extensively searched, incorporating all identified keywords, index terms, and Boolean operators. Keywords included "instrumental delivery" or "forceps vaginal delivery" or "operative vaginal delivery" or forceps assisted birth and "vacuum assisted vaginal delivery" or "vacuum extraction" or "rotational vaginal delivery" or "ventouse cup" and "labouring mother" or "labouring woman" or "female" or "vaginal birth" and "maternal morbidity" or "neonatal morbidity" or "birth injuries" or "birth canal injuries." Additionally, we manually reviewed the reference lists of selected articles and assessments to identify further studies meeting the criteria. In addition, the reference lists from the chosen articles were checked to find more relevant research. The identified references were downloaded into Mendeley, where duplicates were deleted. Two writers then independently assessed the articles’ titles and abstracts to discover possibly relevant material for inclusion. The selected research articles were then subjected to a full-text screening, which was performed individually by two authors. Any differences were settled by consulting with a third author to establish an agreement on the final decision.

The inclusion criteria for articles in this systematic review were as follows: (a) studies focused on the use of obstetric forceps and vacuum extractors, (b) study designs comparing the efficacy of these tools with a control group (vacuum extractor only) and involving randomized trials, and (c) the primary outcome being the incidence of perineal tear. Articles were excluded if they: (a) were systematic reviews, observational, or in vitro studies, (b) had incomplete data, or (c) included other comparators such as normal vaginal delivery and caesarean section.

Two authors separately retrieved data from each included study, including the first author's surname, publication year, country of origin, study design, participant age, number of participants, use of obstetric forceps or vacuum extractor, and the incidence of perineal tears expressed numerically. Any conflicts were handled through discussion or by involving a third investigator.

The included studies’ methodological validity and risk of bias were evaluated using the Cochrane's standard guidelines and Cochrane risk tool, which covers aspects such as randomization, participant concealment, blinding, and selective outcome reporting.^(10)^ When necessary, questions were directed to the authors of the studies. Only research articles of high methodological quality were included in the review. Disputes were settled amicably. Based on the retrieved data, a risk graph and risk summary were created.

The primary outcome of interest reported in the included studies was the number of cases of perineal tear. Secondary outcomes included vaginal injury, cephalohematoma, maternal anaesthetic requirements, and failure to achieve vaginal delivery with the intended instrument.

The meta-analysis which determines the efficacy of obstetrics forceps and vacuum extractor to assist during vaginal delivery was carried out using the "metabin" functions in R software (version 4.2.3). The data were presented as the number of incidents and evaluated using risk differences. A random-effects and common effect model with 95% confidence intervals (CI) was used. The I² test were used to investigate heterogeneity.

The systematic review and meta-analysis comprised a limited number of articles (<10), hence the primary outcome was not examined for publication bias.

## Results

### Study search result

**Figure 1:**
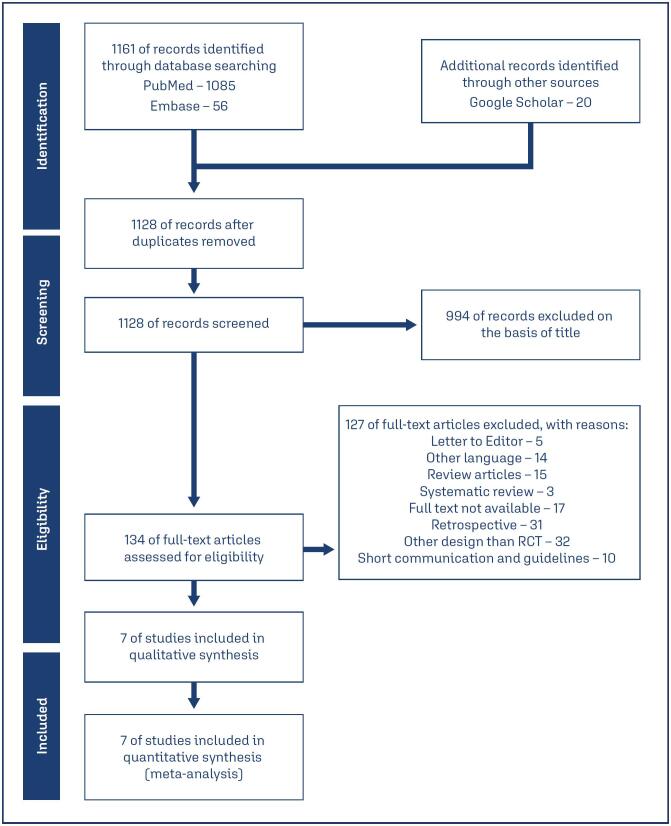
PRISMA flow diagram

We used PRISMA standards for searching and selecting studies that matched the inclusion criteria (Figure 1). Electronic databases revealed a total of 1,161 records. Of these, 33 were duplicates owing to database overlap, leaving 1,128 references to be reviewed. Based on their titles, 994 records were deemed irrelevant and excluded. The complete texts of the remaining 134 publications were reviewed for eligibility, and 127 were excluded for not meeting the inclusion criteria. Ultimately, seven studies met the pre-specified inclusion criteria and were included in the systematic review and meta-analysis.

### Study characteristics

Chart 1 summarizes the features of the included studies. There were a total seven studies with 2,299 participants, 1,173 were in the obstetrical forceps group and 1,126 were in the vacuum extractor group. In terms of nations represented, two studies each were conducted in the United States and the United Kingdom, while one study each was conducted in India, Sri Lanka, and South Africa. The participants’ average ages ranged from 21.11 to 27 years in the intervention group (obstetric forceps) and from 20.56 to 28 years in the control (vacuum extractor group). Each of the studies were randomized controlled trials. all research^(11-17)^ reported the primary outcome, which was the number of perineal tears. Secondary outcomes in this trial included vaginal injury,^(11-14,16)^ cephalohematoma,^(12-14,16,17)^ maternal anaesthetic requirements,^(11,13-16)^ and failure to achieve vaginal delivery with the intended instrument.^(12-17)^

**Chart 1:** Summary table of studies included in the systematic review (efficacy of obstetrics Forceps Vs. vacuum extractor)

Author, year of study	Country of study	Study design	Age in Years (Mean ± SD)		Total No. of participants (n)		Primary objective (Perineal tear)		Secondary objectives Intervention (Obstetric Forceps)/ Control (vacuum extractor)
			Intervention: Obstetric Forceps	Control: vacuum extractor	Intervention: Obstetric Forceps	Control: vacuum extractor	Intervention: Obstetric Forceps (n)	Control: vacuum extractor (n)	
Arya et al. (2001)^(11)^	United States of America	RCT	23.4 ± 2.4 (only primiparous)	21.6 ± 3.2 (only primiparous)	90	75	13	04	• Vaginal injury (88/62) • Episiotomy (90/73) • Maternal anaesthetic requirement (65/59)
Bofill et al. (1996)^(12)^	United States of America	RCT	21.11 ± 5.0	20.56 ± 5.0	315	322	90	38	• Vaginal injury (185/157) • Episiotomy (209/97) • Cephalhematoma (19/37) • Failure to achieve vaginal delivery with assigned instruments (35/21) • Estimated blood loss (347.6±139/355.5 ±116)
Johanson et al. (1993)^(13)^	South Africa	RCT	25.7 ± 5	26.1 ± 5	311	296	297	275	• Vaginal injury (121/84) • Cephalhematoma (08/27) • Scalp and facial injury (45/53) • Maternal anaesthetic requirement (307/277) • Failure to achieve vaginal delivery with assigned instruments (32/45) • Estimated blood loss (337±208/297±184 ml)
Shekhar et al. (2013)^(14)^	India	RCT	24.4 ± 5.6	25.2 ± 5.8	50	50	02	00	• Vaginal injury (27/10) • Cephalhematoma (02/06) • Maternal anaesthetic requirement (50/47) • Failure to achieve vaginal delivery with assigned instruments (00/05) • Estimated blood loss (337/234 ml)
Sultan et al. (1998)^(15)^	United Kingdom	RCT	27	28	17	27	14	13	• Maternal anaesthetic requirement (15/19) • Failure to achieve vaginal delivery with assigned instruments (01/03)
Vacca et al. (1983)^(16)^	United Kingdom	RCT	Median age 24 Years	Median age 24 Years	152	152	24	09	• Vaginal injury (10/05) • Cephalhematoma (08/14) • Maternal anaesthetic requirement (151/150) • Failure to achieve vaginal delivery with assigned instruments (23/29)
Weerasekera e Premaratne (2002^)(17)^	Sri Lanka	RCT	Not reported	Not reported	238	204	04	02	• Cephalhematoma (02/12) • Failure to achieve vaginal delivery with assigned instruments (16/28)

RCT - randomized control trial, SD - Standard deviation, N - Number of participants

### Perineal tear

**Figure 2:**
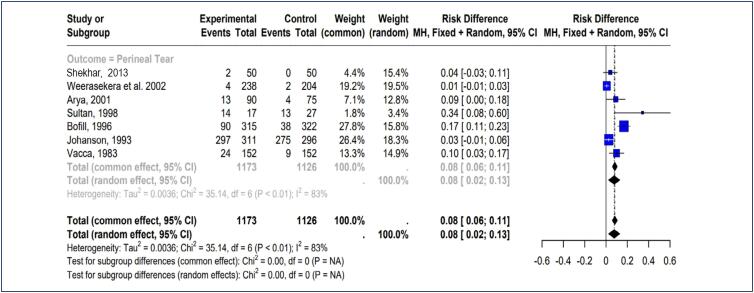
Forest plot of comparison: Forceps versus Ventouse, primary outcome: Perineal tear

A meta-analysis was carried on seven trials,^(11-17)^ which reported perineal tear with 785 patients randomized for obstetric forceps and vacuum extractor group (Obstetric Forceps= 444; vacuum extractor=341). This finding suggests that forceps have a significantly slightly higher risk of perineal tear (444/1173) compared to vacuum extractor (341/1126) shows that the forceps lowers the probability of perineal tear when compared to the vacuum as evidenced by the negative risk difference values (Risk Difference = 0.08, 95% CI 0.02–0.13) and overall combined effect. However, considerable heterogeneity (I² = 83%, p <0.01) indicates variety in impact sizes among trials (Figure 2).

### Vaginal Injury

**Figure 3:**
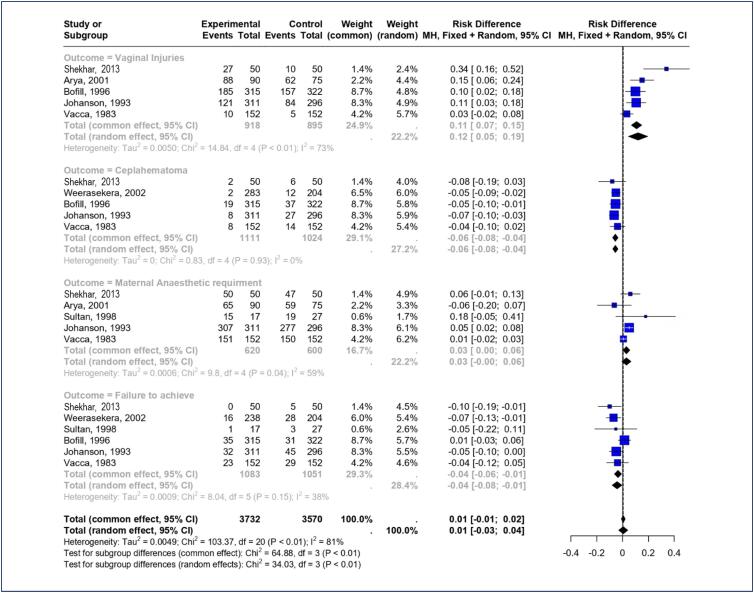
Forest plot of comparison: Forceps versus Ventouse, secondary outcome: Vaginal injury, cephalohematoma, maternal anaesthetic requirements, failure to achieve vaginal delivery with the intended instrument

Five trails^(11-14,16)^ including a total of 749 patients in Obstetric Forceps (431) and vacuum extractor group (318) reported vaginal injury as a study outcome. The forceps’ overall effect shows a somewhat higher risk of vaginal injuries, with a 0.12 [0.05–0.19] overall risk difference and confidence intervals that typically fail to cross the no-effect line. There is substantial variability among the studies (I² = 73%, p <0.01), suggesting diversity in the effect size (Figure 3).

### Cephalohematoma

Forest plot (Figure 3) shows the results of the meta-analysis of five trials^(12-14,16,17)^ reported Cephalohematoma as one of the study outcomes after interventions. The number of cephalohematoma were high in the patients delivered with vacuum extractor (96/1024) than the forceps delivery (39/1111). A negative RD of −0.06 [-0.08, −0.04] indicates that the forceps appears to lower the risk of cephalhematoma. The heterogeneity for this outcome is low (I² = 0%), indicating consistent findings across studies. The fact that the confidence intervals for all of the studies do not cross the no-effect line reinforces the treatment's efficacy in lowering this risk.

### Maternal anaesthetic requirements

Five trails^(11,13-16)^ including a total of 1140 patients (Obstetric Forceps 588/620 and vacuum extractor 552/600) reported maternal anaesthetic requirements as an outcome measure. The effect of the forceps on the need for maternal anaesthetic requirement is minimal, with a risk difference of 0.03 [0.00, 0.06]. The confidence intervals for the studies are relatively close to zero, and the heterogeneity is moderate (Chi² = 9.8; df = 4; P = 0.04; I² = 59%), suggests moderate variability among the studies (Figure 3).

### Failure to achieve vaginal delivery with the intended instrument

Six trails^(12-17)^ included 248 patients (Obstetric Forceps 107/1083 and vacuum extractor 141/1051) reported failure to achieve vaginal delivery with the intended instrument as an outcome. There is a small advantage for Ventouse compared to Forceps, with a combined overall risk difference of −0.04 [-0.08, −0.01]. The heterogeneity statistics is relatively low (Tau² = 0.009; Chi² = 8.09; df = 5; I² = 38%), show minimal variation among included studies (Figure 3).

### Risk of Bias

The authors meticulously reviewed and assessed the research for potential biases. Table 1 summarizes the Cochrane risk assessment, as well as the methodology used. The completeness of the review procedure ensured comprehensive assessment, which increased the credibility of the findings. This in-depth assessment sheds light on the biases prevalent in the included studies as shown in figures 4A and 4B.

**Figure 4A:**
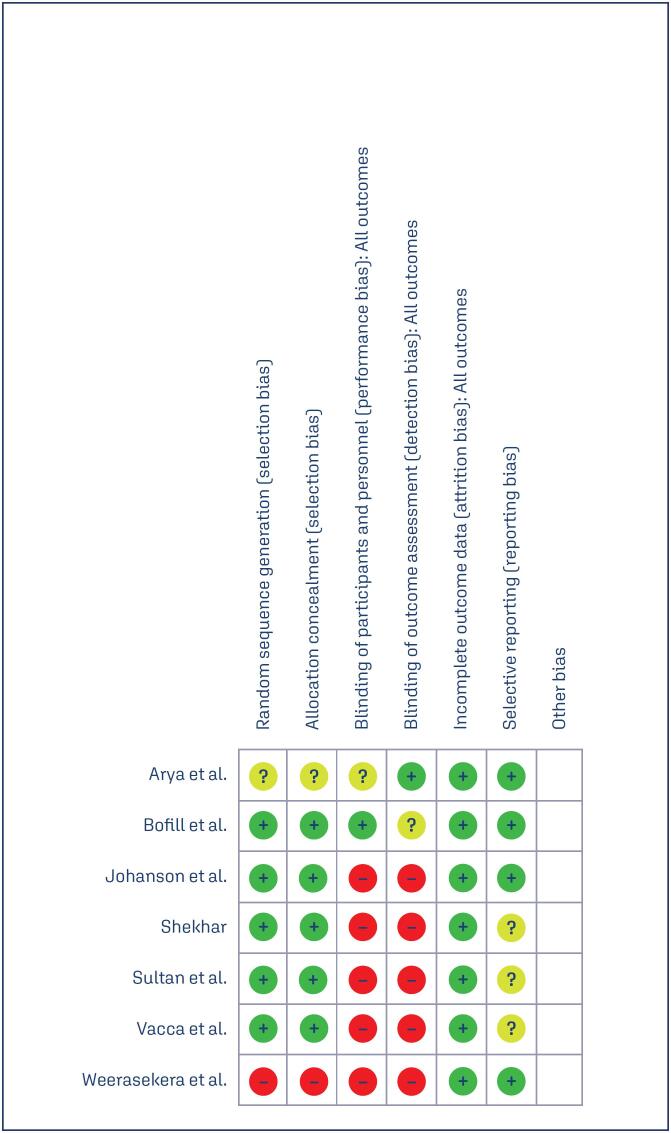
Risk of bias summary: review authors’ judgements about each risk of bias item for each included study

**Figure 4B:**
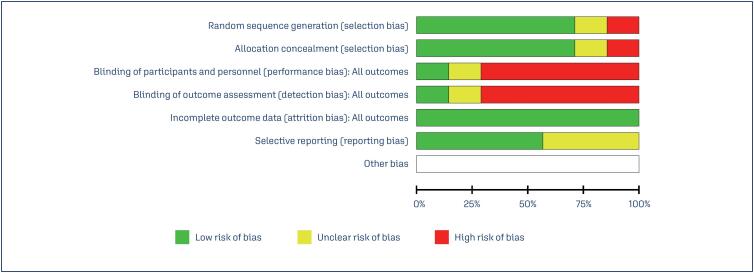
Risk of bias graph: review authors’ judgements about each risk of bias item presented as percentages across all included studies

## Discussion

This systematic review and meta-analysis intended to determine the efficacy and safety of obstetric forceps and vacuum extractors to assist with vaginal birth. Our research findings show that these two instrumental techniques produce significantly different maternal and neonatal outcomes.

The primary outcome of this review was the prevalence of perineal tears. Our meta-analysis of seven studies revealed that the use of obstetric forceps is associated with a higher risk of perineal tears compared to vacuum extractors. This finding is consistent with earlier research, which demonstrates that while forceps are helpful at ensuring delivery, as well as they may cause more severe perineal trauma.^(18-22)^ The heterogeneity demonstrates variability among studies, possibly may be due to variations in study demographics, expertise and skill levels of doctors can significantly impact the effectiveness and safety of using forceps versus vacuum extractors. along with this, procedural protocols and clinical practices between institutions can also play a major role.

Secondary outcomes provide further insight into the risks and benefits of these instruments. The forceps group had a substantially greater incidence of vaginal injury than the vacuum extractor group. This gives credence to the theory that forceps, despite their precision, might cause greater direct harm to the vaginal tissues.^(19,23,24)^ The risk of cephalohematoma was considerably greater in neonates delivered using vacuum extractors. This is consistent with known complications of vacuum extraction, where the suction mechanism can cause subgaleal haemorrhage.^(21,25-27)^ The assessment of maternal anaesthetic requirements found no significant difference between the two groups. This shows that both instruments require equivalent degrees of anaesthetic, most likely due to the intrusive nature of the operations. However, substantial heterogeneity indicates some variety in anaesthetic administration procedures. Vacuum extractors were somewhat more likely to fail vaginal delivery than forceps. This finding reveals a slight but statistically significant preference for the use of forceps when a quick and precise delivery is required. The minor variations among studies (I² = 38%) indicates that the doctor's ability and experience may possibly impact the outcome.

Obstetric forceps may cause more maternal perineal trauma, vacuum extractors have been linked to a surge in newborn cephalohematomas. Both instruments require equal levels of anaesthetic and have similar rates of successful births. The decision between forceps and vacuum extractors should be based on the unique clinical scenario, taking into account the risks and benefits. Future research should focus on improving the safety profiles of these instruments and developing uniform guidelines to improve maternal and neonatal outcomes.

## Conclusion

This systematic review and meta-analysis demonstrate that the obstetric forceps during vaginal delivery increases the likelihood of perineal tears when compared to vacuum extractors. While forceps can help with birth, they can also cause significant maternal trauma. The significant heterogeneity indicates that study demographics, surgeon experience, and procedural procedures all may have an impact on outcomes. These findings show the need of personalized training and established guidelines for reducing risks and improving the safety and effectiveness of instrumental vaginal births.

## Data availability

: The authors did not make the data from this article available in repositories prior to submission.
